# Uroperitoneum in cattle: Ultrasonographic findings, diagnosis and treatment

**DOI:** 10.1186/s13028-015-0126-y

**Published:** 2015-07-03

**Authors:** Ueli Braun, Karl Nuss

**Affiliations:** Department of Farm Animals, Vetsuisse-Faculty, University of Zurich, Winterthurerstrasse 260, CH-8057 Zurich, Switzerland

**Keywords:** Cattle, Ascites, Uroperitoneum, Bladder rupture, Persistent urachus, Ultrasonography

## Abstract

This review describes causes, clinical signs, metabolic changes in serum and peritoneal fluid, diagnosis and treatment of uroperitoneum. Rupture of the bladder or urachus is the most common cause of uroperitoneum. The main clinical sign is a pear-shaped enlargement of the abdomen accompanied by gradual deterioration in demeanour and appetite. Ultrasonography shows massive accumulation of anechoic abdominal fluid and organs suspended in the fluid. Bladder defects may be seen cystoscopically and the proximal part of a persistent urachus can be explored endoscopically. Abdominocentesis yields light yellow fluid. A peritoneal-to-serum creatinine concentration ratio of 2 or greater is diagnostic of uroperitoneum. Treatment consists of surgical repair of the defect.

**Electronic supplementary material:**

The online version of this article (doi:10.1186/s13028-015-0126-y) contains supplementary material, which is available to authorized users.

## Introduction

Uroperitoneum is accumulation of urine in the peritoneal cavity caused by leakage of urine from the kidneys, ureters, urinary bladder or urethra [1] or from a ruptured persistent urachus [2, 3]. Renal trauma caused by blunt force, entrapment, falls, puncture or accidental trocarisation [4] may rupture the renal capsule leading to leakage of urine into the perirenal tissues. Leakage of urine from a kidney may also occur with hydronephrosis caused by a ureteral obstruction. Retroperitoneal accumulation of urine referred to as urinoma is usually the result of leakage from a traumatised ureter [1]. In male cattle, rupture of the urethra usually leads to subcutaneous urine accumulation with pitting oedema along the ventral abdominal wall (waterbelly) and in the inguinal region [4]. Rupture of the bladder and urachus are the predominant causes of uroperitoneum in female cattle [2, 3]. The goal of this review is to describe the causes, clinical signs, diagnosis and treatment of uroperitoneum in cattle.

### Preparation of the review

The databases PubMed and VetMed Resource for the years 1975 to January 2015 were searched in January 2015 for the keywords cattle, cow, calf, uroperitoneum, bladder, urachus, urachal rupture, bladder rupture, ultrasonography. In addition, the list of references of standard texts (references 1, 4 and 5) were scrutinised for relevant articles. All publications related to the topic of this review were included. There were no publication restrictions.

## Review

### Uroperitoneum caused by bladder rupture

In male cattle, bladder rupture is usually secondary to obstruction of urinary outflow [4], the most common cause of which is urolith-induced urethral obstruction [5]. Urethral strictures caused by injury, necrotising inflammation, surgical procedures such as urethrotomy or castration [4] or urethral compression by a tumour, abscess or haematoma are rare causes of urinary outflow obstruction and bladder rupture [4, 6]. One case report described urethral obstruction, urinary outflow obstruction and eventual bladder rupture due to haematoma formation in the urethral submucosa in a three-month-old bull calf [7]. In another report, a haematoma associated with a comminuted fracture of the first two coccygeal vertebrae led to urethral compression and subsequent bladder rupture in a 16-month-old Limousin bull [6]. Bladder rupture in a four-month-old bull was thought to be caused by infection of the umbilical artery in the neonatal period, which led to necrosis and inflammation of the bladder wall and creation of a diverticulum [8].

**Fig. 1:**
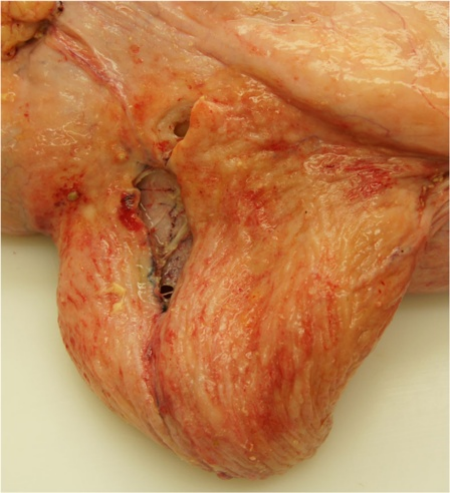
Bladder rupture attributable to dystocia. Bladder rupture attributable to dystocia in a 2.3-year-old Red Holstein cow

**Fig. 2:**
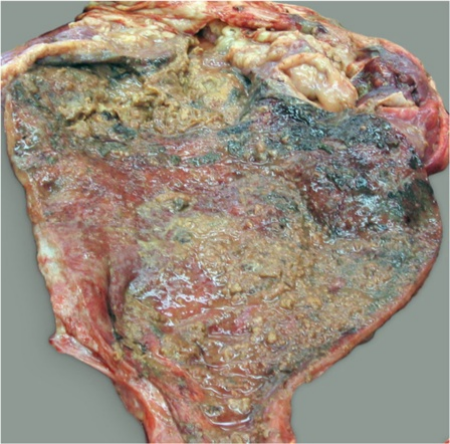
Necrotising cystitis attributable to dystocia. Postmortem view of necrotising cystitis in a 4.5-year-old Simmental cow. This was attributable to dystocia and resulted in bladder rupture. The bladder mucosa is discoloured and thickened, has numerous erosions and ulcerations and is covered with fibrin (from Braun *et al.* [11])

In female cattle, bladder rupture occurs most commonly after dystocia (Fig. 1) [4, 5, 9, 10] but also can result from necrotising cystitis (Fig. 2) [11]. Obstruction of the neck of the bladder by a fibrin cast led to bladder rupture in a six-year-old Ayrshire cow [12]. The cause of bladder rupture in a one-year-old Holstein heifer could not be determined [13], and in another heifer of the same age and breed, the formation of adhesions after surgical removal of a urachal abscess was thought to be the cause of bladder rupture [14]. Perforating bladder injuries have been reported after breeding accidents, accidental introduction of an insemination pipette into the bladder, careless catheterisation and sadism [4]. Small injuries may heal spontaneously without the occurrence of uroperitoneum [4].

### Uroperitoneum caused by urachal rupture

Immediately after umbilical cord rupture, the urachus retracts into the abdominal cavity toward the apex of the bladder, and its peritoneal covering gives rise to the vesicoumbilical ligament. The distinct conical vestige of the urachus at the apex of the bladder is referred to as urachal umbilicus in the German veterinary literature [15]. Urachal anomalies result from partial or complete failure of urachal involution, which has been described extensively in humans [16]. Failure of complete urachal obliteration results in a persistent urachus, which is accompanied by urine dribbling from the urachus during or after urination. A partially patent urachus may persist as a bladder diverticulum, which is patent toward the bladder but obliterated toward the umbilicus. A subcutaneous urachal diverticulum can also occur in calves with a defect in the linea alba and a persistent urachus that is patent from the bladder to the umbilicus [17]. An umbilical fistula occurs when the urachus is obliterated toward the bladder but remains patent toward the umbilicus. A urachal cyst may arise when the middle section of the urachus remains patent and both ends obliterate [18]. Reports of anomalies of the urachus and bladder reflect the wide spectrum of changes associated with abnormal urachal involution.

**Fig. 3:**
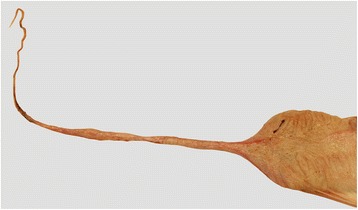
Persistent urachus. Intact urinary bladder and persistent urachus from a 3.3-year-old Braunvieh x Brown Swiss cow

Incomplete urachal involution may result in spontaneous urachal rupture but the aetiology of this remains obscure (Fig. 3). It has been described in calves [8, 19], heifers [20] and mature cattle [2, 3, 21] (Table 1). Several reports on congenital urethral obstruction in calves with subsequent urachal rupture and uroperitoneum have been published [19, 22, 23]. Rupture of the urachal diverticulum caused by obstruction of the urethra was described in five bulls with urolithiasis [21]. Multiple ruptures of a persistent urachus were reported in a yearling bull [20], and rupture of a urachus that was patent toward the bladder but obliterated distally was described in four cows [2, 3]. Two of the latter cases [2] involved a ruptured diverticulum at the apex of the bladder, which in all likelihood represented a urachal remnant. However, it is also conceivable that the diverticula were caused by urinary outflow obstruction of the bladder. This has been described in humans [24], in which diverticula always occur at a congenitally compromised site of the bladder such as the apex. Abnormal urachal involution is inherited in humans and is thought to have a hereditary component in cattle [20]. The cases of abnormal urachal involution seen in our clinic were all Brown Swiss-Braunvieh cross cattle, which supports the notion of an inherited anomaly. Because most urachal problems manifest in young animals, it is remarkable that the cases of urachal rupture seen at our clinic occurred in cattle aged 1.5 to 5 years. This phenomenon also occurs in people, albeit rarely; ruptured urachal cyst was reported in a 63-year-old man [25] and an 80-year-old woman [26], and spontaneous rupture of a urachal diverticulum was described in a 38-year-old man [27]. The aetiology of urachal rupture in adult humans is not known but it is conceivable that increased tenesmus associated with urinary outflow obstruction leads to rupture of a previously asymptomatic urachus because of increased pressure in the bladder. The urachal wall is much thinner than the bladder wall in cattle and therefore predisposed to rupture [17]. It is suspected that a persistent urachus has a limited capacity to grow and dilate in cattle and therefore is prone to rupture because of stretching and thinning [2].

**Table 1:** Urachal anomalies in cattle with ruptured urachus

Authors	Breed	Sex	Age	Pregnant	Lesion
Baxter *et al.* [20]	Beef	M	1.5 years	NA	1
Bell *et al.* [8]	Limousin	M	0.3 years	NA	2
Braun *et al.* [2]	BV x BS	F	5.0 years	5 months	1
	BV x BS	F	1.5 years	No	1
	BV x BS	F	1.5 years	4 months	2
	BV x BS	F	3.0 years	No	1
	BV x BS	F	4.0 years	No	2
Braun *et al.* [3]	BV x BS	F	2.0 years	7 months	1
Braun, not published	BV x BS	F	3.1 years	7 months	1
Edwards *et al.* [23]	Charolais x Brangus	I	4 days	NA	1
Hylton and Trent [22]	Charolais	F	4 days	NA	1
Marques *et al.* [21]	5 bulls of different breeds	M	5 – 7 years	NA	2
Nikahval and Khafi [19]	Holstein Friesian	F	10 days	NA	3

BV x BS Braunvieh x Brown Swiss

Sex: M Male, F Female, I Intersex (freemartin)

NA Not applicable

Lesion 1: Rupture of persistent urachus; urachus was persistent near the bladder and involuted near the umbilicus

Lesion 2: Ruptured diverticulum, which was most likely a urachal remnant, at the pole of the bladder

Lesion 3: Urethral obstruction, persistent urachus, uroperitoneum

### Clinical signs of uroperitoneum

**Fig. 4:**
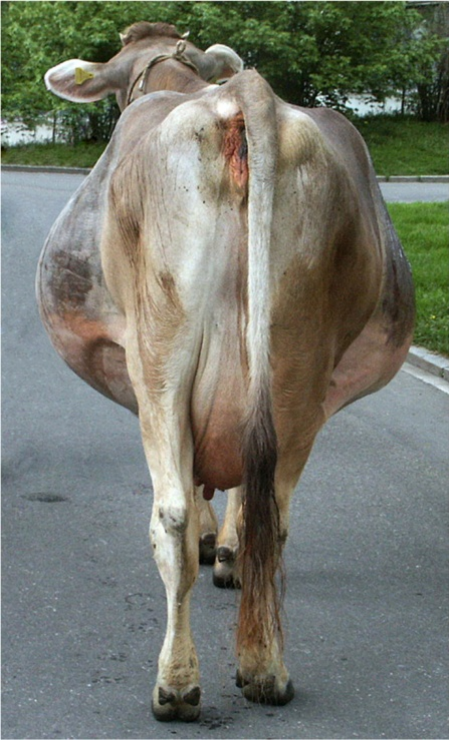
Pear-shaped abdomen - Posterior view. Posterior view of a four-year-old Braunvieh x Brown Swiss cow with a pear-shaped abdomen caused by rupture of a persistent urachus

**Fig. 5:**
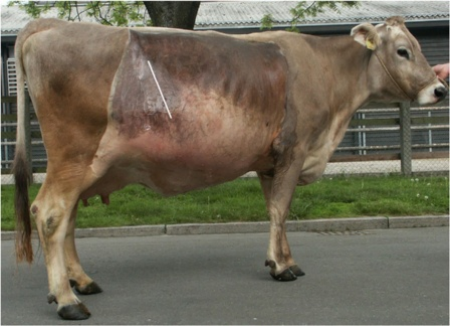
Abdominal distension - Lateral view. Lateral view of abdominal distension of the cow shown in Fig. 4. The skin incision for surgical correction is marked in white (or: as a white line)

There are several reports of clinical signs of urachal [2, 3, 8, 20] and bladder rupture [4, 9–11] in cattle. The classical clinical sign of bladder rupture is a pear-shaped abdomen (Fig. 4) attributable to accumulation of urine in the peritoneal cavity (Fig. 5). The abdominal wall is soft and sloshing sounds may be elicited on succussion. Although there are no distinct signs of pain following bladder rupture, uroperitoneum is accompanied by tachycardia, enophthalmus, reduced skin turgor, ruminal atony and gradual deterioration in demeanour and appetite [2]. Intraabdominal fluid may preclude transrectal palpation of the rumen as well as the bladder because it is empty or contains little urine, and sloshing of fluid is felt instead. One study found that there was very little manure in the rectum of several cows with uroperitoneum and sometimes fibrin and blood were found, which was unexpected because this is more typical of ileus [2]. Urination behaviour usually does not allow a diagnosis of uroperitoneum because it may be normal or absent or there may be stranguria [2, 3]. An old diagnostic method for bladder rupture involved the infusion of 30 ml of 1 % methylene blue into the bladder followed by abdominocentesis; blue discolouration of the collected fluid was considered diagnostic of bladder rupture [20]. A historical method used to diagnose ruptured bladder in people entailed the injection of air into the bladder and simultaneous auscultation of the abdomen for sounds created by air escaping from the bladder into the abdomen [28].

### Metabolic changes associated with uroperitoneum

**Fig. 6:**
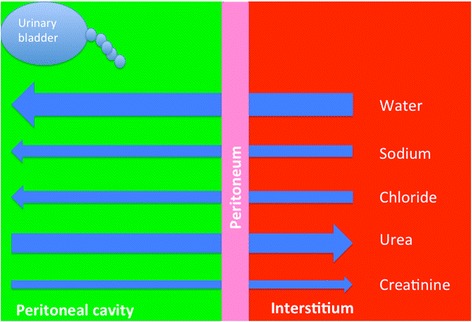
Osmotic and metabolic changes in cattle with uroperitoneum. Schematic representation of osmotic and metabolic changes in peritoneal and interstitial fluid in cattle with uroperitoneum (modified from Donecker and Bellamy [29])

Metabolic changes associated with uroperitoneum in cattle have been described by several authors [29–31]. The changes are based on diffusion of solutes and osmosis of water across the peritoneum, which constitutes a selectively permeable membrane. Essentially, uroperitoneum is accompanied by the following changes (Fig. 6): Firstly, water moves down an osmotic gradient from the interstitial space into the peritoneal fluid because urine osmolality is two to three times higher than the osmolality of interstitial fluid. This results in dehydration with reduced skin turgor, enophthalmus and haemoconcentration. Secondly, sodium and chloride diffuse into the peritoneal fluid because the concentration of these electrolytes is lower in urine than in blood. This results in hyponatraemia and hypochloridaemia; however, these changes were not seen in calves after experimental bladder rupture [31]. Thirdly, urea and creatinine pass down a concentration gradient from the peritoneal fluid into the extravascular space. Creatinine is a larger molecule and therefore diffuses more slowly and at a lower rate than urea. Although these changes are characteristic of uroperitoneum, they are not pathognomonic [29]. The only diagnostic test for uroperitoneum is measuring creatinine concentration in serum and peritoneal fluid. A peritoneal-to-serum creatinine concentration ratio of 2 or greater is diagnostic of uroperitoneum [5].

**Fig. 7:**
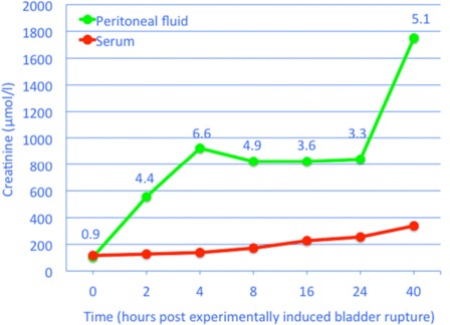
Creatinine concentrations after experimental bladder rupture. Creatinine concentrations in serum and peritoneal fluid in the first 40 h after experimental bladder rupture in 5 Holstein Friesian calves (modified from Wilson and MacWilliams [31])

Metabolic changes associated with uroperitoneum were studied in pre-ruminant calves for 40 h after experimental bladder rupture [31]. Creatinine concentration increased from 115 to 341 μmol/l in serum and from 106 to 1,752 μmol/l in peritoneal fluid (Fig. 7). The peritoneal-to-serum creatinine concentration ratio was 0.9 at time 0, 4.4 at 4 hrs and ranged from 3.3 to 6.6 until the end of the study period.

### Haematological and serum biochemical findings in cattle with uroperitoneum

Increased urea and creatinine serum concentrations are typical biochemical changes in cattle with uroperitoneum [13, 14, 29–31] and were seen in all cases referred to our clinic (Table 2). Furthermore, most cattle had haemoconcentration and increased haematocrit because of loss of water into the peritoneal space. There was mild leukocytosis or leukopenia, the potassium concentration was slightly increased or decreased and sodium and chloride concentrations were slightly lower than or within the reference intervals.

**Table 2:** Results of haematological analysis in 8 cows with uroperitoneum

Variable (reference interval)	Cow 1^1^	Cow 2^1^	Cow 3^1^	Cow 4^1^	Cow 5^1^	Cow 6^2^	Cow 7^3^	Cow 8^4^
Urea (2.7-5.7 mmol/l)	16.1	33.7	19.9	18.5	26.1	18.5	37.5	9.0
Creatinine (88–133 μmol/l)	260	608	451	251	350	319	727	194
Haematocrit (25–37 %)	ND	46	48	45	42	45	39	32
Total leukocyte count (3.9-9.1 x 10^3^/μl)	ND	12.8	9.2	6.4	8.7	3.9	10.9	8.7
Potassium (3.8-5.3 mmol/l)	4.1	5.4	5.4	5.3	3.6	4.1	5.0	4.2
Sodium (143–157 mmol/l)	148	141	144	133	136	138	141	146
Chloride (98–109 mmo/l)	89	92	99	82	84	91	96	107

^1^From Braun *et al.* [2]

^2^From Braun *et al.* [2]

^3^From Braun *et al.* [3]

^4^From Braun unpublished (cow with ruptured persistent urachus, see Table 1)

### Urinary findings in cattle with urachal or bladder rupture

Although urination may be absent, urine can be collected by catheterisation in most cows with urachal or bladder rupture because the bladder is not completely empty. In a study of five cows with urachal/bladder rupture, urine could be collected with a catheter in all but one [2]. In that study, the urine was grossly normal in three cows, and a urine test strip showed haematuria in two cows [2]. Uroperitoneum is accompanied by isosthenuria indicating that large amounts of urine are produced in an attempt at eliminating waste from the blood.

### Ultrasonography

The entire abdomen is examined ultrasonographically from both sides. In addition, transrectal ultrasonographic examination is carried out and the penile urethra is scanned for concrement in male animals. The examination is done in the standing non-sedated animal, but calves can also be examined in a lying position. The skin is clipped and cleaned with alcohol and conductive gel is applied. Linear or convex transducers with a frequency of 3.5 or 5 MHz are best suited but abnormalities close to the skin also can be assessed with a 7.5-MHz transducer. The abdomen is first examined on the right side from caudal to cranial. The transducer is placed at the paralumbar fossa and then moved ventrally to the midline. This is repeated in a cranial direction toward the last rib and then the last two intercostal spaces are examined in a similar fashion. The examination is repeated on the left side in an analogous manner. The bladder and urethra are scanned transrectally with the transducer directed ventrally and the caudal part of the left kidney is examined with the transducer directed dorsally. In male animals, the penile urethra is examined between the scrotum and prepuce [32]. The abdominal organs normally occupy the entire abdominal cavity and are separated from each other and the peritoneum by capillary spaces, which contain very small amounts of serous fluid to lubricate the surface of tissues. The capillary spaces are not normally visible ultrasonographically but can be imaged when they enlarge as a result of fluid accumulation or other disease processes. Likewise, the omentum and mesentery are difficult to visualise ultrasonographically in healthy ruminants but are easily seen when separated by fluid. The high fat content of these structures increases sound reflection. The organ contours usually are smooth and echoic deposits with or without fluid inclusions are considered abnormal. The fluid between organs is assessed for amount and echogenicity; the latter can range from anechoic to echoic and may appear homogeneous or heterogeneous. If the fluid is an exudate, echoic inflammatory sediment may be seen at the lowest point accompanied by a hypoechoic supernatant. Strands of fibrin often can be seen running in a spider web-like fashion between organs or between an organ and the parietal peritoneum.

**Fig. 8:**
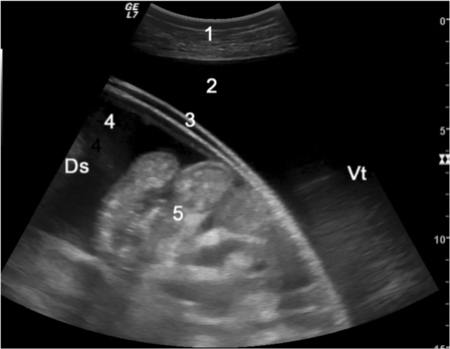
Ultrasonogram of the abdomen in a cow with uroperitoneum. Ultrasonogram of the abdomen obtained from the right flank using a 5.0-MHz convex transducer in a 3.1-year-old Braunvieh x Brown Swiss cow with uroperitoneum caused by rupture of a persistent urachus. 1: Abdominal wall, 2: Extraomental accumulation of anechoic fluid (uroperitoneum), 3: Greater omentum, 4: Intraomental accumulation of anechoic fluid (uroperitoneum), 5: Small intestine, Ds: Dorsal, Vt: Ventral

**Fig. 9:**
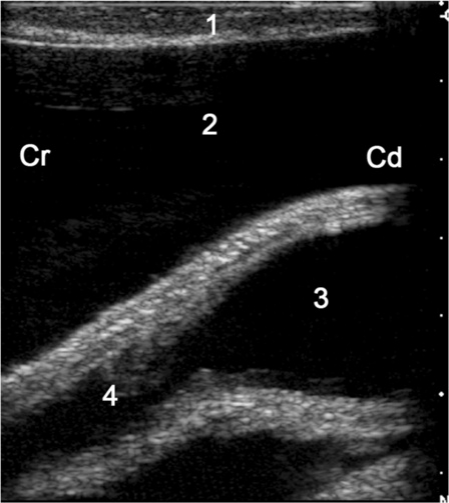
Ultrasonogram of the bladder in a cow with rupture of a persistent urachus. Ultrasonogram of the bladder obtained transrectally using a 5.0-MHz linear transducer in a 4-year-old Braunvieh x Brown Swiss cow with uroperitoneum caused by rupture of a persistent urachus. The persistent urachus is visible at the cranial pole of the bladder. 1: Rectum, 2: Anechoic fluid (uroperitoneum) surrounding the bladder, 3: Bladder, 4: Persistent urachus, Cr: Cranial, Cd: Caudal (from Braun *et al.* [2])

Ultrasonograms of uroperitoneum show massive fluid accumulation involving the entire abdomen and organs that appear suspended in the fluid (Fig. 8). Transrectal ultrasonographic examination may identify a ruptured bladder, which may be collapsed and flaccid and contain little or no urine or contain varying amounts of urine provided that fibrin has sealed the defect [32]. If a persistent urachus exerts traction on the bladder, the urine-filled transition from the bladder to the urachus can be seen ultrasonographically (Fig. 9). Sometimes the bladder is surrounded by urine. A completely empty bladder may not be seen. Male cattle with urethral rupture have pitting oedema along the ventral abdomen, which is considered diagnostic of this condition [32].

### Abdominocentesis in cattle with uroperitoneum

**Fig. 10:**
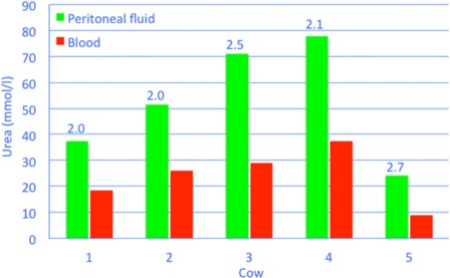
Peritoneal-to-serum urea concentration ratios in cows with uroperitoneum. Peritoneal-to-serum urea concentration ratios in 5 Braunvieh x Brown Swiss cows with uroperitoneum caused by rupture of a persistent urachus. The numbers on top of the green columns indicate the ratios for each cow

**Fig. 11:**
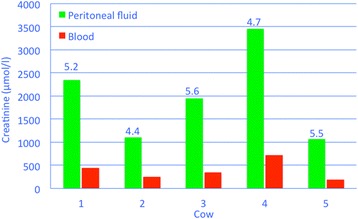
Peritoneal-to-serum creatinine concentration ratios in cows with uroperitoneum. Peritoneal-to-serum creatinine concentration ratios in 5 Braunvieh x Brown Swiss cows with uroperitoneum caused by rupture of a persistent urachus. The numbers on top of the green columns indicate the ratios for each cow

Abdominocentesis is carried out under ultrasonographic guidance after clipping the hair and disinfecting and anaesthetising the skin. The recovered fluid is light yellow or colourless and clear and only rarely smells of urine [2]. Specific gravity and protein concentration usually are very low and urea and creatinine concentrations very high. Cows with uroperitoneum referred to our clinic had peritoneal-to-serum urea concentration ratios ranging from 2 to 2.7 (Fig. 10); the peritoneal urea concentration ranged from 24.8 to 77.8 mmol/l and the serum urea concentration from 9 to 37.5 mmol/l. The peritoneal-to-serum creatinine concentration ratio ranged from 4.4 to 5.6 (Fig. 11); the peritoneal creatinine concentration ranged from 1’112 to 3’453 μmol/l and the serum creatinine concentration from 251 to 727 μmol/l.

### Cystoscopy

**Fig. 12:**
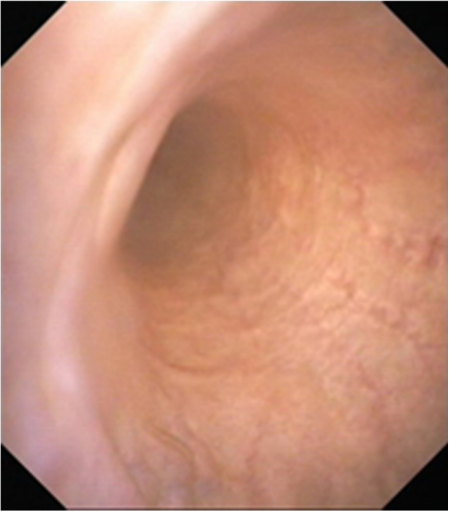
Cystoscopy in a cow with ruptured urachus. Cystoscopic view of a persistent urachus in a 2-year-old Braunvieh x Brown Swiss cow with ruptured urachus (from Braun *et al.* [3])

Cystoscopy is a useful technique for the detection of bladder rupture or a persistent urachus [3, 11]. A flexible video endoscope with a diameter of about 9 mm and a working length of at least 100 cm is ideal for viewing the rupture in the bladder wall. With urachal rupture, the bladder is stretched longitudinally and the endoscope can be introduced into the urine-filled urachus [3] (Fig. 12); however, we have been unable to visualise the site of urachal rupture because our endoscope was not long enough.

### Prognosis

Bladder rupture caused by dystocia has a poor prognosis because the bladder wall often is severely contused and compromised [11]. In contrast, the bladder musculature and mucosa are not or only minimally affected with urachal rupture and therefore uroperitoneum caused by urachal rupture usually has a favourable prognosis [2].

### Surgical treatment of uroperitoneum

**Fig. 13:**
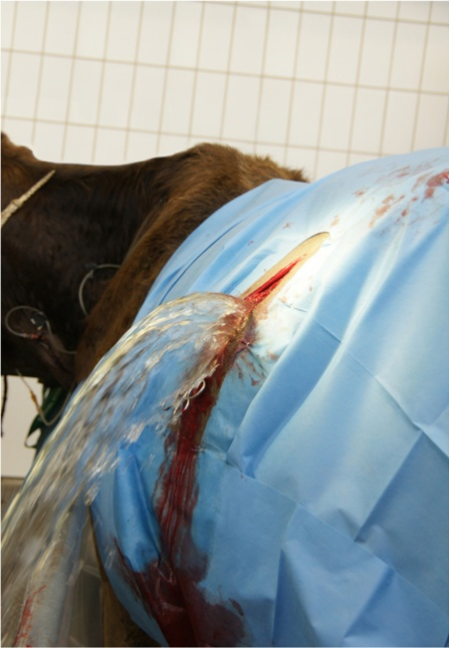
Release of urine from the abdomen. Intraoperative release of urine in a 2-year-old Braunvieh x Brown Swiss cow with uroperitoneum caused by rupture of a persistent urachus

Surgical correction of a ruptured persistent urachus causing uroperitoneum in heifers and cows is usually carried out in the left or right flank with the animal standing. The skin incision is made in a caudodorsal to cranioventral direction starting about 5 cm ventral to the transverse process of the 5th lumbar vertebra and extending to a point about 5 cm caudal to the costal arch at the level of the transition of the last rib to its costal cartilage (Fig. 5). The external oblique abdominal muscle is incised but the internal oblique abdominal muscle is split using blunt dissection in the direction of the muscle fibres. This incision direction facilitates exploration of the caudal region of the abdominal cavity and exteriorisation of the bladder through the incision. Large amounts of urine, often in excess of 100 litres, spontaneously escape through the incision upon opening the abdominal cavity (Fig. 13) as shown in Additional file 1. The abdomen is then explored and the surgeon determines whether the bladder or urachus is ruptured.

A ruptured urachus usually is not completely detached but can be identified as a tissue strand between the pole of the bladder and the internal umbilical ring. The urachus can be broken manually at its attachment to the umbilicus or it may be cut close to the umbilicus using blunt/blunt scissors. Cutting with scissors is safe because adjacent organs and the omentum are suspended in urine and thus lifted off the ventral abdominal wall. The remaining urine is evacuated and the abdominal cavity rinsed with isotonic saline solution. The free end of the urachus and the bladder usually can be pulled into the incision and examined for ruptures [17]. Persistent urachus is associated with elongation of the bladder, which aids in exteriorisation of the bladder. The bladder is retracted with the aid of stay sutures or clamps and the pole of the bladder with the attached urachus is resected. The bladder is closed in two layers, a continuous suture oversewn by an inverting suture [33, 34] or two inverting sutures [17]. The abdominal wall is closed after lavage of the muscle layers to reduce the risk of contamination.

The method of treatment of bladder rupture depends of the location of the wall defect. Dorsal tears may heal spontaneously if a Foley catheter is placed transcutaneously or through the urethra to drain the urine [33]. Ventral tears usually require surgery via caudal flank or ventral midline coeliotomy using local or general anaesthesia. Bladder distension using saline solution aids in localising the wall defect and in ensuring secure closure after surgery. Bladder surgery is difficult in large cows because exteriorisation of the bladder and visualisation of the defect can be a problem and much of the suturing may have to be done blindly. Alternatively, laparoscopic or laparoscopic-assisted surgical techniques may be used to close the bladder defect [35]. The suturing technique is analogous to that described for urachal tears [34]. If an uroperitoneum is caused by leakage of urine from an ureter or a kidney, unilateral nephrectomy may be performed.

Postoperative treatment includes procaine penicillin or amoxicillin for 5 to 7 days and a nonsteroidal antiinflammatory drug such as flunixin meglumine for 3 days. Intravenous fluid therapy consisting of daily infusion of 10 l of a NaCl-glucose solution (50 g glucose and 9 g sodium chloride/l) for 3 to 5 days is recommended.

### Convalescence

**Fig. 14:**
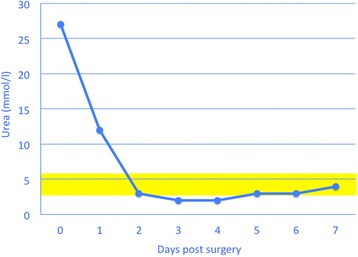
Serum urea concentration after surgical treatment of ruptured urachus. Serum urea concentration after surgical treatment of ruptured persistent urachus in a 4-year-old Braunvieh x Brown Swiss cow. The yellow bar represents the reference interval for urea (2.7 to 5.7 mmol/l)

**Fig. 15:**
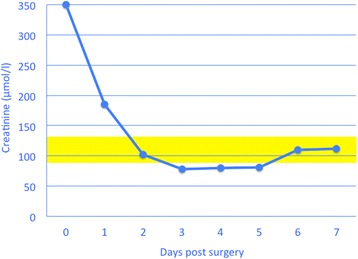
Serum creatinine concentration after surgical treatment of ruptured urachus. Serum creatinine concentration after surgical treatment of ruptured persistent urachus in a 4-year-old Braunvieh x Brown Swiss cow. The yellow bar represents the reference interval for creatinine (88 to 133 μmol/l)

The rapid improvement in demeanour and appetite and normalisation of serum biochemical values after surgical repair of urachal or bladder rupture is impressive in cattle. After about two days, most cattle appear clinically normal and serum concentrations of urea and creatinine are within reference intervals (Figs. 14 & 15).

## Conclusions

Diagnosis of uroperitoneum is not always straightforward because other diseases that result in accumulation of intraabdominal fluid must be ruled out. In most cases, uroperitoneum can be diagnosed based on the results of ultrasonography and abdominocentesis and calculation of the peritoneal-to-serum creatinine concentration ratio. The cause of uroperitoneum often can be determined and the condition corrected surgically.

## Additional file

Additional file 1:
**Video showing drainage of urine from the abdomen.** Surgical incision of the abdominal wall in the right flank of a 3.1-year-old, 7 months pregnant Braunvieh x Brown Swiss cow with uroperitoneum caused by rupture of a persistent urachus. More than 100 l of urine is released from the abdomen.
